# *N*-Benzyl-2,3,4,5,6-penta­fluoro­benz­amide

**DOI:** 10.1107/S1600536810043345

**Published:** 2010-10-31

**Authors:** Arto Valkonen, Tanja Lahtinen, Kari Rissanen

**Affiliations:** a Nanoscience Center, Department of Chemistry, University of Jyväskylä, PO Box 35, FIN-40014 University of Jyväskylä, Finland

## Abstract

In the title compound, C_14_H_8_F_5_NO, the dihedral angle between the planes of the penta­fluoro­phenyl and phenyl rings is 18.34 (5)°. An inter­molecular N—H⋯O hydrogen bond between the amide groups connects these mol­ecules to form an infinite chain through the crystal structure. One weak intermolecular C—H⋯O contact and one π–π interaction [centroid–centroid distance = 3.772 (3) Å] are also involved in crystal structure stabilization between the phenyl rings.

## Related literature

For related structures, see: An & Rhee (2003 ▶); Cockroft *et al.* (2007 ▶); Forbes *et al.* (2001 ▶); Liu *et al.* (2007 ▶); Qadeer *et al.* (2007 ▶); Zhang & Zhang (2008 ▶). For anion⋯π inter­actions, see: Albrecht *et al.* (2010 ▶); Lahtinen & Rissanen (2007 ▶); Müller *et al.* (2010 ▶).
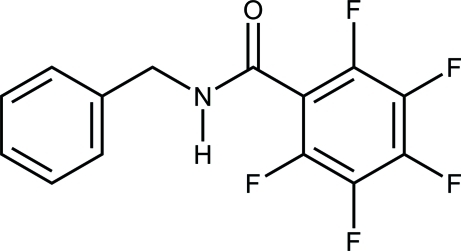


## Experimental

### 

#### Crystal data

C_14_H_8_F_5_NO
*M* *_r_* = 301.21
Monoclinic, 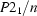

*a* = 7.1649 (2) Å
*b* = 22.9090 (5) Å
*c* = 7.5363 (1) Å
β = 99.205 (2)°
*V* = 1221.08 (5) Å^3^
*Z* = 4
Mo *K*α radiation
μ = 0.16 mm^−1^
*T* = 123 K
0.40 × 0.28 × 0.26 mm

#### Data collection

Bruker Nonius KappaCCD with APEXII detector diffractometer
4246 measured reflections
2152 independent reflections
1891 reflections with *I* > 2σ(*I*)
*R*_int_ = 0.016

#### Refinement

*R*[*F* ^2^ > 2σ(*F* ^2^)] = 0.033
*wR*(*F* ^2^) = 0.086
*S* = 1.06
2152 reflections
193 parameters
1 restraint
H atoms treated by a mixture of independent and constrained refinement
Δρ_max_ = 0.20 e Å^−3^
Δρ_min_ = −0.18 e Å^−3^

### 

Data collection: *COLLECT* (Bruker, 2008 ▶); cell refinement: *DENZO-SMN* (Otwinowski & Minor, 1997 ▶); data reduction: *DENZO-SMN*; program(s) used to solve structure: *SIR2004* (Burla *et al.*, 2005 ▶); program(s) used to refine structure: *SHELXL97* (Sheldrick, 2008 ▶); molecular graphics: *ORTEP-3* (Farrugia, 1997 ▶) and *Mercury* (Macrae *et al.*, 2008 ▶); software used to prepare material for publication: *SHELXL97*.

## Supplementary Material

Crystal structure: contains datablocks global, I. DOI: 10.1107/S1600536810043345/bt5380sup1.cif

Structure factors: contains datablocks I. DOI: 10.1107/S1600536810043345/bt5380Isup2.hkl

Additional supplementary materials:  crystallographic information; 3D view; checkCIF report

## Figures and Tables

**Table 1:** Hydrogen-bond geometry (Å, °)

*D*—H⋯*A*	*D*—H	H⋯*A*	*D*⋯*A*	*D*—H⋯*A*
N8—H8⋯O1^i^	0.88 (1)	2.01 (1)	2.875 (2)	171 (2)
C5—H5⋯O1^ii^	0.95	2.37	3.276 (2)	158

Symmetry codes: (i) 
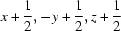
; (ii) 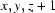.

**Fig. 1.**
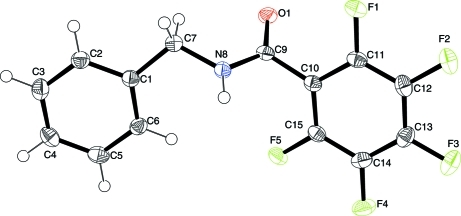
View of the molecule of the title compound showing the atom-labelling scheme. Displacement ellipsoids are drawn at the 50% probability level. H atoms are represented by circles of arbitrary size.

**Fig. 2.**
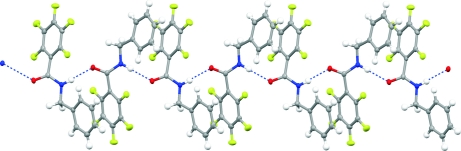
Part of the infinite chain formed by N—H···O hydrogen bonds in the crystal of the title compound.

**Fig. 3.**
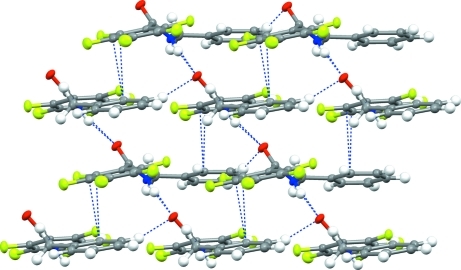
Packing diagram showing the N—H···O, C—H···O and π–π contacts.

## supplementary crystallographic information

### Comment

The title compound is synthesized by classical amide formation reaction between amine (benzylamine) and carboxylic acid halide (2,3,4,5,6-pentafluorobenzoylchloride). The molecule of this secondary amide is not planar and contains two aromatic ring systems (Fig. 1), the other one being electron poor due to electron-withdrawing force of connected F atoms. This pentafluorophenyl moiety has recently been found to be an excellent halogen···π contact acceptor for halide and polyhalide anions in similar ammonium salt structures (Albrecht *et al.*, 2010; Müller *et al.*, 2010) and also acceptor for C═O···C(aromatic) anion···.π-type contacts (Lahtinen & Rissanen, 2007). The pentafluorophenyl ring is found to be inclined to phenyl ring by 18.34 (5)°. The C10/C9/N8/O1 amide group is more significantly inclined to pentafluorophenyl ring by 56.95 (4)° and to phenyl ring by 56.21 (4)°, as also observed, for example, with few substituted *N*-phenyl-2,3,4,5,6-pentafluorobenzamides (Cockroft *et al.*, 2007) and *N*-Benzyl-4,5-dimethoxy-2-nitrobenzamide (Qadeer *et al.*, 2007).

The intermolecular interactions of the title compound include one N—H···O, one C—H···O (Table 1) and one π–π contacts. The N—H···O hydrogen bonds connect the molecules to form infinite chain in (*x* + 1/2, -*y* + 1/2, *z* + 1/2) direction (Fig. 2), where every second molecule is in same orientation and every second is rotated 180° on the direction of *b* axis. Similar chain was obtained, for example,with *N*-Benzyl-4-phenylbenzamide (An & Rhee, 2003). These chains are connected to each other by one C—H···O (Table 1) and one π–π contacts (Fig. 3), the latter having centroid-to-centroid distance of 3.772 (3) Å and closest C···C distance of 3.327 (3) Å. These distances are slightly longer than in the structure of *N*-(2-pyridyl)-2,3,4,5,6-pentafluorobenzamide (Forbes *et al.*, 2001). The C═O···C(aromatic) anion···.π-type contact, found from the related structure of *N*-[1-(silatran-1-yl)propyl]pentaflurobenzamide (Lahtinen & Rissanen, 2007), seems to be in this case forced by nearby N—H···O contact. Fluorines F3 and F4 (Fig. 1) show distance 2.920 (2) Å to F3 and F4 of the neighbouring molecule in (-*x*, -*y*, -*z* + 1) direction, but this contact is most probably too weak to be significant in crystal stabilization.

### Experimental

Benzylamine (184 mg, 1.72 mmol) and triethylamine (470µl, 3.44 mmol) were mixed in dry DCM under inert atmosphere (Ar). The reaction mixture was cooled (ice–salt bath) and 2,3,4,5,6-pentafluorobenzoylchloride (240µl, 1.72 mmol) in dry DCM was added dropwise to the reaction mixture. After addition the reaction mixture was stirred in ice–salt bath for 1 h and in room temperature for additional 20 h. The reaction mixture was washed twice with water and organic layer was dried and evaporated to yield white solid product. For the single-crystal X-ray analysis the crude product was recrystallized from CHCl_3_ yielding colourless needles.

### Refinement

All H atoms were visible in electron density maps, but those bonded to C were ideally positioned and allowed to ride on their parent atoms at C—H distances of 0.95 Å (aromatic) and 0.99 Å (methylene), with *U*_iso_(H) of 1.2 times *U*_eq_(C). The N—H proton were found in the electron density map and was refined with a distance restraint [N—H = 0.88 (2) Å], and *U*_iso_(H) = 1.2 times *U*_eq_(N) was used.

### Crystal data

**Table:**

C_14_H_8_F_5_NO	*F*(000) = 608
*M**_r_* = 301.21	*D*_x_ = 1.638 Mg m^−^^3^
Monoclinic, *P*2_1_/*n*	Mo *K*α radiation, λ = 0.71073 Å
*a* = 7.1649 (2) Å	Cell parameters from 3094 reflections
*b* = 22.9090 (5) Å	θ = 0.4–28.3°
*c* = 7.5363 (1) Å	µ = 0.16 mm^−^^1^
β = 99.205 (2)°	*T* = 123 K
*V* = 1221.08 (5) Å^3^	Block, colourless
*Z* = 4	0.40 × 0.28 × 0.26 mm

### Data collection

**Table:**

Bruker Nonius KappaCCD with APEXII detector diffractometer	1891 reflections with *I* > 2σ(*I*)
Radiation source: fine-focus sealed tube	*R*_int_ = 0.016
graphite	θ_max_ = 25.0°, θ_min_ = 2.9°
Detector resolution: 9 pixels mm^-1^	*h* = −8→8
φ and ω scans	*k* = −27→27
4246 measured reflections	*l* = −8→8
2152 independent reflections	

### Refinement

**Table:**

Refinement on *F*^2^	Primary atom site location: structure-invariant direct methods
Least-squares matrix: full	Secondary atom site location: difference Fourier map
*R*[*F*^2^ > 2σ(*F*^2^)] = 0.033	Hydrogen site location: inferred from neighbouring sites
*wR*(*F*^2^) = 0.086	H atoms treated by a mixture of independent and constrained refinement
*S* = 1.06	*w* = 1/[σ^2^(*F*_o_^2^) + (0.0393*P*)^2^ + 0.498*P*] where *P* = (*F*_o_^2^ + 2*F*_c_^2^)/3
2152 reflections	(Δ/σ)_max_ < 0.001
193 parameters	Δρ_max_ = 0.20 e Å^−^^3^
1 restraint	Δρ_min_ = −0.18 e Å^−^^3^

### Special details

**Table:**

Geometry. All s.u.'s (except the s.u. in the dihedral angle between two l.s. planes) are estimated using the full covariance matrix. The cell s.u.'s are taken into account individually in the estimation of s.u.'s in distances, angles and torsion angles; correlations between s.u.'s in cell parameters are only used when they are defined by crystal symmetry. An approximate (isotropic) treatment of cell s.u.'s is used for estimating s.u.'s involving l.s. planes.
Refinement. Refinement of *F*^2^ against ALL reflections. The weighted *R*-factor *wR* and goodness of fit *S* are based on *F*^2^, conventional *R*-factors *R* are based on *F*, with *F* set to zero for negative *F*^2^. The threshold expression of *F*^2^ > 2σ(*F*^2^) is used only for calculating *R*-factors(gt) *etc*. and is not relevant to the choice of reflections for refinement. *R*-factors based on *F*^2^ are statistically about twice as large as those based on *F*, and *R*- factors based on ALL data will be even larger.

### Fractional atomic coordinates and isotropic or equivalent isotropic displacement parameters (Å^2^)

**Table:**

	*x*	*y*	*z*	*U*_iso_*/*U*_eq_	
F1	0.11992 (14)	0.19154 (4)	−0.04946 (11)	0.0315 (2)	
F2	0.17000 (14)	0.07495 (4)	−0.05119 (12)	0.0350 (3)	
F3	0.14683 (15)	0.01169 (4)	0.24882 (14)	0.0379 (3)	
F4	0.06766 (15)	0.06526 (4)	0.54949 (12)	0.0363 (3)	
F5	0.00325 (13)	0.18090 (4)	0.54897 (11)	0.0277 (2)	
O1	−0.10620 (15)	0.27306 (4)	0.12572 (13)	0.0260 (3)	
N8	0.12659 (19)	0.28716 (5)	0.36339 (16)	0.0232 (3)	
H8	0.218 (2)	0.2705 (7)	0.437 (2)	0.028*	
C1	0.1073 (2)	0.37470 (6)	0.55552 (19)	0.0207 (3)	
C2	0.1399 (2)	0.43426 (7)	0.5826 (2)	0.0240 (3)	
H2	0.1652	0.4579	0.4856	0.029*	
C3	0.1359 (2)	0.45945 (7)	0.7488 (2)	0.0278 (4)	
H3	0.1570	0.5002	0.7649	0.033*	
C4	0.1011 (2)	0.42535 (7)	0.8916 (2)	0.0277 (4)	
H4	0.0983	0.4425	1.0059	0.033*	
C5	0.0703 (2)	0.36600 (7)	0.8668 (2)	0.0262 (4)	
H5	0.0474	0.3424	0.9648	0.031*	
C6	0.0729 (2)	0.34078 (7)	0.6995 (2)	0.0226 (3)	
H6	0.0509	0.3001	0.6835	0.027*	
C7	0.1041 (2)	0.35043 (6)	0.3685 (2)	0.0262 (4)	
H7A	−0.0174	0.3612	0.2935	0.031*	
H7B	0.2068	0.3689	0.3149	0.031*	
C9	0.0197 (2)	0.25426 (6)	0.24216 (18)	0.0197 (3)	
C10	0.0625 (2)	0.18984 (6)	0.25080 (18)	0.0197 (3)	
C11	0.1035 (2)	0.16115 (7)	0.09949 (19)	0.0224 (3)	
C12	0.1315 (2)	0.10168 (7)	0.0975 (2)	0.0249 (4)	
C13	0.1202 (2)	0.06950 (7)	0.2499 (2)	0.0258 (4)	
C14	0.0806 (2)	0.09667 (7)	0.4021 (2)	0.0253 (4)	
C15	0.0511 (2)	0.15619 (7)	0.40118 (19)	0.0215 (3)	

### Atomic displacement parameters (Å^2^)

**Table:**

	*U*^11^	*U*^22^	*U*^33^	*U*^12^	*U*^13^	*U*^23^
F1	0.0414 (6)	0.0350 (5)	0.0190 (5)	−0.0003 (4)	0.0075 (4)	0.0023 (4)
F2	0.0382 (6)	0.0351 (5)	0.0326 (5)	0.0004 (4)	0.0089 (4)	−0.0129 (4)
F3	0.0420 (6)	0.0199 (5)	0.0515 (6)	0.0005 (4)	0.0070 (5)	−0.0003 (4)
F4	0.0463 (6)	0.0303 (5)	0.0315 (5)	−0.0016 (4)	0.0039 (4)	0.0131 (4)
F5	0.0343 (5)	0.0314 (5)	0.0176 (4)	−0.0017 (4)	0.0050 (4)	−0.0009 (4)
O1	0.0271 (6)	0.0248 (6)	0.0223 (5)	−0.0011 (5)	−0.0072 (5)	0.0037 (4)
N8	0.0258 (7)	0.0204 (7)	0.0203 (6)	0.0035 (5)	−0.0057 (5)	−0.0006 (5)
C1	0.0169 (7)	0.0222 (8)	0.0215 (7)	0.0018 (6)	−0.0015 (6)	0.0001 (6)
C2	0.0239 (8)	0.0241 (8)	0.0232 (8)	−0.0008 (6)	0.0016 (6)	0.0022 (6)
C3	0.0255 (8)	0.0233 (8)	0.0333 (9)	−0.0014 (7)	0.0006 (7)	−0.0045 (7)
C4	0.0249 (9)	0.0354 (9)	0.0227 (8)	0.0027 (7)	0.0034 (6)	−0.0067 (7)
C5	0.0217 (8)	0.0350 (9)	0.0222 (8)	0.0016 (7)	0.0046 (6)	0.0041 (6)
C6	0.0209 (8)	0.0201 (7)	0.0256 (8)	0.0005 (6)	0.0001 (6)	0.0019 (6)
C7	0.0345 (9)	0.0212 (8)	0.0212 (8)	0.0008 (7)	−0.0004 (6)	0.0009 (6)
C9	0.0191 (8)	0.0242 (8)	0.0158 (7)	−0.0019 (6)	0.0025 (6)	0.0024 (6)
C10	0.0148 (7)	0.0243 (8)	0.0186 (7)	−0.0017 (6)	−0.0020 (5)	−0.0001 (6)
C11	0.0193 (8)	0.0288 (8)	0.0184 (7)	−0.0028 (6)	0.0006 (6)	0.0028 (6)
C12	0.0188 (8)	0.0291 (8)	0.0262 (8)	−0.0014 (6)	0.0017 (6)	−0.0074 (7)
C13	0.0212 (8)	0.0192 (8)	0.0355 (9)	−0.0010 (6)	0.0000 (7)	−0.0002 (6)
C14	0.0229 (8)	0.0260 (8)	0.0256 (8)	−0.0031 (6)	−0.0002 (6)	0.0074 (6)
C15	0.0183 (8)	0.0269 (8)	0.0183 (7)	−0.0018 (6)	−0.0008 (6)	−0.0014 (6)

### Geometric parameters (Å, °)

**Table:**

F1—C11	1.3419 (17)	C3—H3	0.9500
F2—C12	1.3439 (18)	C4—C5	1.385 (2)
F3—C13	1.3381 (18)	C4—H4	0.9500
F4—C14	1.3393 (17)	C5—C6	1.390 (2)
F5—C15	1.3418 (17)	C5—H5	0.9500
O1—C9	1.2313 (17)	C6—H6	0.9500
N8—C9	1.3287 (19)	C7—H7A	0.9900
N8—C7	1.4596 (19)	C7—H7B	0.9900
N8—H8	0.876 (14)	C9—C10	1.507 (2)
C1—C6	1.388 (2)	C10—C15	1.384 (2)
C1—C2	1.394 (2)	C10—C11	1.388 (2)
C1—C7	1.512 (2)	C11—C12	1.377 (2)
C2—C3	1.383 (2)	C12—C13	1.378 (2)
C2—H2	0.9500	C13—C14	1.374 (2)
C3—C4	1.385 (2)	C14—C15	1.380 (2)
			
C9—N8—C7	121.84 (13)	N8—C7—H7B	108.8
C9—N8—H8	118.6 (11)	C1—C7—H7B	108.8
C7—N8—H8	119.4 (11)	H7A—C7—H7B	107.7
C6—C1—C2	118.69 (14)	O1—C9—N8	124.59 (14)
C6—C1—C7	122.99 (13)	O1—C9—C10	119.63 (13)
C2—C1—C7	118.28 (13)	N8—C9—C10	115.78 (12)
C3—C2—C1	120.92 (14)	C15—C10—C11	117.21 (14)
C3—C2—H2	119.5	C15—C10—C9	122.86 (13)
C1—C2—H2	119.5	C11—C10—C9	119.79 (13)
C2—C3—C4	120.05 (14)	F1—C11—C12	118.15 (13)
C2—C3—H3	120.0	F1—C11—C10	120.00 (14)
C4—C3—H3	120.0	C12—C11—C10	121.84 (14)
C3—C4—C5	119.56 (14)	F2—C12—C11	120.61 (14)
C3—C4—H4	120.2	F2—C12—C13	119.90 (14)
C5—C4—H4	120.2	C11—C12—C13	119.49 (14)
C4—C5—C6	120.37 (14)	F3—C13—C14	120.15 (14)
C4—C5—H5	119.8	F3—C13—C12	119.81 (14)
C6—C5—H5	119.8	C14—C13—C12	120.04 (14)
C1—C6—C5	120.40 (14)	F4—C14—C13	119.99 (14)
C1—C6—H6	119.8	F4—C14—C15	120.27 (14)
C5—C6—H6	119.8	C13—C14—C15	119.73 (14)
N8—C7—C1	113.87 (12)	F5—C15—C14	118.18 (13)
N8—C7—H7A	108.8	F5—C15—C10	120.08 (13)
C1—C7—H7A	108.8	C14—C15—C10	121.69 (14)
			
C6—C1—C2—C3	−0.8 (2)	F1—C11—C12—F2	−1.4 (2)
C7—C1—C2—C3	177.02 (14)	C10—C11—C12—F2	179.65 (13)
C1—C2—C3—C4	0.7 (2)	F1—C11—C12—C13	178.39 (13)
C2—C3—C4—C5	0.0 (2)	C10—C11—C12—C13	−0.6 (2)
C3—C4—C5—C6	−0.5 (2)	F2—C12—C13—F3	−0.3 (2)
C2—C1—C6—C5	0.3 (2)	C11—C12—C13—F3	179.87 (14)
C7—C1—C6—C5	−177.44 (14)	F2—C12—C13—C14	−179.91 (14)
C4—C5—C6—C1	0.4 (2)	C11—C12—C13—C14	0.3 (2)
C9—N8—C7—C1	135.84 (15)	F3—C13—C14—F4	−0.3 (2)
C6—C1—C7—N8	−19.9 (2)	C12—C13—C14—F4	179.26 (13)
C2—C1—C7—N8	162.42 (14)	F3—C13—C14—C15	−179.20 (14)
C7—N8—C9—O1	−0.4 (2)	C12—C13—C14—C15	0.4 (2)
C7—N8—C9—C10	178.57 (13)	F4—C14—C15—F5	−2.2 (2)
O1—C9—C10—C15	−121.13 (16)	C13—C14—C15—F5	176.69 (13)
N8—C9—C10—C15	59.85 (19)	F4—C14—C15—C10	−179.71 (13)
O1—C9—C10—C11	54.5 (2)	C13—C14—C15—C10	−0.8 (2)
N8—C9—C10—C11	−124.55 (15)	C11—C10—C15—F5	−176.90 (12)
C15—C10—C11—F1	−178.80 (13)	C9—C10—C15—F5	−1.2 (2)
C9—C10—C11—F1	5.4 (2)	C11—C10—C15—C14	0.5 (2)
C15—C10—C11—C12	0.1 (2)	C9—C10—C15—C14	176.25 (14)
C9—C10—C11—C12	−175.70 (14)		

### Hydrogen-bond geometry (Å, °)

**Table:**

*D*—H···*A*	*D*—H	H···*A*	*D*···*A*	*D*—H···*A*
N8—H8···O1^i^	0.88 (1)	2.01 (1)	2.875 (2)	171 (2)
C5—H5···O1^ii^	0.95	2.37	3.276 (2)	158

Symmetry codes: (i) *x*+1/2, −*y*+1/2, *z*+1/2; (ii) *x*, *y*, *z*+1.
